# Comparing Neighbors and Friends in Age-Related Network Changes

**DOI:** 10.1093/geronb/gbae108

**Published:** 2024-06-29

**Authors:** Matthijs Kalmijn

**Affiliations:** Netherlands Interdisciplinary Demographic Institute (NIDI)/KNAW, Hague, The Netherlands; Department of Sociology, University of Groningen, Groningen, The Netherlands

**Keywords:** Cohort changes, Divorce, Support, Life-course changes, Retirement, Social integration, Socioeconomic status, Weak ties, Widowhood

## Abstract

**Objectives:**

To assess how the role of neighbors and friends in people’s networks changes with age and how this is affected by cohort, marriage, employment, and socioeconomic status. The hypothesis is that for most aspects of the network, friends lose “importance” as people become older, with neighbors gradually becoming more dominant in the nonkin network.

**Methods:**

Data are used for people aged 55–90 between 1999 and 2019 from the Swiss Household Panel (*N* = 5,585). A total of 4 network aspects were measured: size, contact, practical support, and emotional support. Measures for neighbors and friends were compared and analyzed with fixed-effects and hybrid-effects regression models on person-year observations.

**Results:**

The sizes of both network segments declined with age but more strongly for friends than neighbors. Contact with friends was stable but contact with neighbors increased. Support from friends declined whereas support from neighbors was stable. Direct comparisons revealed that the relative share of neighbors vis-à-vis friends increased as people age. Friends were more common and supportive vis-à-vis neighbors for divorced and widowed people than for married people, but this gap declined with age. The share of neighbors increased with retirement, especially for men. The share of neighbors vis-à-vis friends was also larger for people with less income and education and this gap did not change with age.

**Discussion:**

In the nonkin part of older adults’ networks, proximity eventually becomes dominant. This finding is interpreted in terms of rising needs, greater opportunity for local contact, and friend mortality risks, all favoring the neighbor segment of the network.

The importance of neighbors and friends in old age has been relatively well-established in the research literature. Although many older people rely on their partner and, to a lesser extent, their adult children for socioemotional and practical support, the role of nonkin has been recognized early on (Cantor, 1979; Rosow, 1970). According to socioemotional selectivity theory, people’s emotional needs are stronger as they age, and for this reason, friends become more important (Carstensen, 1992; Lang et al., 1998). Next to emotional needs, older people increasingly need practical support and this makes neighbors, being geographically close, a relevant part of the network in old age as well (Wellman & Wortley, 1990). Studies have found that good relationships with neighbors and friends are generally beneficial for well-being and health (Cramm et al., 2013; Greenfield & Reyes, 2015; Luo, 2023; Turner et al., 2022).

Although many studies have analyzed changes in social networks and social contacts across the life course (see following), few have directly compared neighbors and friends. Using longitudinal data collected in Switzerland between 1999 and 2019, this study tracks individual changes in the relative importance of neighbors and friends for people aged 55–90. The main goal is to describe age-based changes for four network indicators, each time directly contrasting neighbors and friends: the number of network members, the amount of contact, the degree of practical support, and the degree of emotional support. Which type of tie is more important in old age, how does this change as people age, and how do these changes depend on well-known network determinants, particularly birth cohort, marriage, employment, and socioeconomic status? The term “importance” is used to denote differences in the size of networks, the amount of contact, and expected support. These are relevant aspects for comparing the two subnetworks but such a comparison does not necessarily correspond to what people themselves would say if we would ask them about which part of the network they would perceive as more important.

A growing number of studies have tracked changes in social networks over time using panel data (Cornwell et al., 2014; Kalmijn, 2012; Klaus & Schnettler, 2016; Schwartz & Litwin, 2018; Shaw et al., 2007). However, most dynamic analyses have not focused on comparing neighbors and friends. Some studies have analyzed changes in separate network segments. Studies often find a decline in the number of friends and a parallel decline in contact with friends (Klaus & Schnettler, 2016; Shaw et al., 2007; Stevens & van Tilburg, 2011) but there are also contrary findings (Martire et al., 1999). One study that combined within- and between-person variation of neighbor support in Europe showed a positive association between age and support received from neighbors (Seifert & König, 2019). Cross-sectional analyses have also examined the role of age and neighborhood ties in samples of older adults. Some find positive associations with age (Shaw, 2005), but others find no association after controlling for other life course factors, particularly retirement (Cornwell et al., 2008). Because age effects can be biased by cohort effects, panel data are more suitable for examining age-related changes in the relative importance of neighbors and friends.

## Background and Hypotheses

Neighbors and friends are often regarded as fundamentally different. For example, according to Wenger, “friendship is based on choice and shared interest being primarily an expressive relationship; while neighboring is based on proximity and is primarily an instrumental relationship” (Wenger, 1990, p. 149). Friendships are often considered strong ties whereas neighbors are the prototypical weak ties (Volker & Flap, 2007). The two types of ties play a role in debates about postmodernization, where due to the process of individualization, local and in part “given” relationships such as church members and neighbors are believed to have declined in relevance over time, whereas voluntarily chosen ties such as friends have become more important (Adams & Allan, 1998; Allan, 1998; Fischer, 1982, 2011).

Some authors have also emphasized their similarities. Neighbors and friends comprise the main component of the nonkin network, especially in old age where colleagues and schoolmates play a limited role. Moreover, neighbors are to some extent voluntary. One can choose where to live and one can avoid contact with neighbors. The key difference remains in geography, however. Although neighbors can become friends and friends can live close, friends will be more geographically dispersed for most people. For this reason, comparing neighbors and friends in old age is relevant more generally as it provides clues about the possibly persisting importance of proximity in personal relationships in society (Wiles et al., 2012).

Although traditionally, it was believed that neighbors are “third in line” behind family and friends, recently, such notions have been criticized by pointing to neighbors’ importance beyond the practical domain and their beneficial effects on individual well-being (Greenfield & Reyes, 2015), a notion also voiced in recent theorizing about the importance of “aging in place” (Gardner, 2011). The general hypothesis motivating this contribution is that the importance of neighbors versus friends increases with age (*H1a*). The hypothesis can be derived from a preference-constraint model of social networks which argues that changes in the size and composition of networks depend on the preferences people have to interact with certain alters, and on the opportunities they have to meet these alters in their day-to-day lives (Kalmijn, 2012; Marsden, 1990; Mollenhorst et al., 2008). Preferences are shaped by various concerns, including support needs, homophily, and personality. Opportunities depend on a variety of factors, including the composition of the social context, group size, mobility, and functional associations such as memberships and employment. Based on this general perspective, three reasons motivate the hypothesis.

First, opportunities for contact with neighbors and friends in old age depend on mortality patterns. Mortality affects the friendship network more strongly than the neighbor network. Studies have shown that the death of a close friend is a common occurrence in the lives of older adults (Cornwell & Laumann, 2018). Neighbors will die too but will be replaced and new, younger neighbors can take their place in the network. Although there is age segregation in neighborhoods (Das Gupta & Wong, 2022), in Europe, old-age or retirement communities are less common than in the United States (Glass & Skinner, 2013), making neighbor networks younger than friendship networks on average (Uhlenberg & Gierveld, 2004).

Second, opportunities and preferences work together in health concerns. A decline in health and physical mobility among older people will make the more geographically dispersed parts of the network more vulnerable (Simonsick et al., 1998). Because face-to-face contact remains an important way to maintain networks, travel restrictions will limit the amount of contact with friends and may ultimately lead to a decline in the number of friends compared to neighbors. Practical needs are not only increased by health problems but also by age-related declines in the ability to maintain day-to-day tasks (Scheel-Hincke et al., 2020). Changing support needs increase preferences for proximate contact and benefit the neighbor part of the network because proximity is key for practical support.

A third and related aspect of opportunity lies in employment patterns. Because of retirement, older people spend more time in their neighborhood, automatically increasing opportunities to build ties with neighbors. More opportunity for contact is associated with stronger neighbor relationships (Volker & Flap, 2007). Studies often point to a decline in the number of weaker ties after retirement due to the loss of colleagues and associated network members (Kauppi et al., 2021). This loss can be compensated by increased neighborhood ties after retirement, leading to a possible neutral effect of retirement on network size in general.

When zooming in on specific aspects of the network, there are competing views about which network segment will change most—and for whom—and these largely depend on the changing preferences people have for contact as they age. Socioemotional selectivity theory argues that friends will become more important with age. People’s emotional needs are stronger as they age and for this reason, “interaction with a select group of significant others becomes increasingly valuable” (Carstensen, 1992, p. 332). In line with this, studies have shown diminished well-being benefits with a larger number of contacts for older people (Luo et al., 2022). Hence, the prediction is that the emotional strength of friendships vis-à-vis neighbors will gain importance with age, even if the relative number of friends in the network declines (*H1b*). This hypothesis is partly in contrast with our first hypothesis (*H1a*) but only for one aspect of the network (emotional support).

Next to a description of change, this study addresses several other relevant factors for understanding how the importance of neighbors and friends changes, in particular cohort, marital status, employment, and socioeconomic status (SES). These factors may have implications for how the importance of neighbors and friends changes across the life course.

First, we consider cohort differences. So far, little is known about how the relative importance of neighbors versus friends has changed across cohorts. For the general population, there seems to have been no decline in the number of friends that people have, disproving concerns about increasing loneliness (Fischer, 2011). Dutch studies compared cohorts born in the period 1908–1937 and found that older cohorts had fewer friends and smaller networks than recent cohorts of the same age (Stevens & van Tilburg, 2011; Suanet et al., 2013). Changes in technology, transport, and communication are believed to have weakened the importance of local ties across cohorts, thus strengthening the potential of friendships, which are not constrained by proximity (Wellman et al., 2001). Postmodernization scholars further suggest that friends become more important across cohorts because friends can be matched to people’s tastes and preferences and therefore become more important for people’s identity (Adams & Allan, 1998). Moreover, compositional changes across cohorts can make friendships more important, particularly improvements in health, educational expansion, and secularization (Suanet et al., 2013). In broad strokes, these lines of reasoning would suggest that across cohorts, the relative importance of friends vis-à-vis neighbors has increased (*H2*).

Marital status differences have often been studied in connection to social networks (Kalmijn, 2012; Sarkisian & Gerstel, 2016; van Tilburg & Suanet, 2019). Some studies showed that single people, regardless of the reason for being single, receive more support from and have more contact with neighbors than people living with a partner (Sarkisian & Gerstel, 2016; Seifert & König, 2019). Other studies, in contrast, find adverse effects of divorce on neighborhood contact, in line with ideas about the normative sanctioning of divorce on the one hand and marital status homophily in networks on the other hand (Kalmijn & van Groenou, 2005). When focusing on friendships, most studies find that single persons are generally more involved (Kalmijn & van Groenou, 2005; Sarkisian & Gerstel, 2016; Terhell et al., 2004). The relative importance of neighbors vis-à-vis friends is expected to be greater for people who are married than for people who are divorced, widowed, or never married (*H3a*). Marital status differences are expected to decline with age as the demand for practical support eventually grows for single persons as well while aging friends become less able or available (*H3b*).

Changes in employment will also play a role in the size and nature of older people’s networks, in particular retirement. One of the implications of retirement is an increase in available time, combined with a lengthening of the time spent in the neighborhood. As a result, opportunities to maintain ties with neighbors increase. To the extent that friends are also colleagues, opportunities to maintain ties with friends decrease. Studies have found that retirement increases volunteering and memberships, both of which could be more strongly locally oriented (Grunwald et al., 2021; Hank & Stuck, 2008; Van den Bogaard et al., 2014). A cross-sectional U.S. study found that retirement was associated with more socializing in the neighborhood (Cornwell et al., 2008). On top of this, analyses of longitudinal data from SHARE (Survey of Health, Ageing and Retirement) found a decline in the share of friendships in the network after retirement (Comi et al., 2022).

No studies of retirement have looked at both neighbors and friends. In this study, we expect that a transition to nonemployment is associated with an increase in the importance of neighbors versus friends (*H4a*). Because it is plausible that part-time work is less restrictive for developing one’s network, and because women more often work part-time (OECD, 2002), we expect that for women, the transition to nonemployment will have a smaller impact on the relative importance of neighbors vis-à-vis friends than it does for men (*H4b*).

The size and nature of networks depend on SES (Fischer & Beresford, 2015; van Groenou et al., 2006). The classic view is that due to occupational and educational differences, people with a lower SES are more locally oriented than people with a higher SES (Rosow, 1970). Among older people, some studies find that a higher SES is associated with more friends, more contact with them, and more geographically dispersed networks (Arjouch et al., 2005; Fischer & Beresford, 2015; Shaw et al., 2007). At the same time, however, it has been found that older people with a lower SES receive more support from their (nonkin) network (van Groenou et al., 2006). In line with traditional modernization theories, older people with a lower SES receive more support from neighbors (Seifert & König, 2019).

The relative importance of neighbors vis-à-vis friends is expected to be greater for lower-SES groups than for higher-SES groups (*H5a*). Mortality differences may also affect how SES differentials in social networks change. It is well known that mortality is higher among the lower educated and at older ages, this will lead to a faster shrinkage of the number of living friends, without necessarily changing the number of neighbors. As a result, one would expect that the relative share of neighbors increases faster with age for people with a lower SES than for people with a higher SES (*H5b*).

## Method

### Data and Sample

Because of its unique network module, the Swiss Household Panel (SHP) is an important data source on change. The SHP is based on a sample of people aged 14 and over in private households in the noninstitutional resident population of Switzerland (Tillmann et al., 2022). The panel began in 1998 and added refreshment samples (with the same sampling design) to remaining respondents of the original sample in 2004, 2013, and 2020. There are now 23 waves of data. Attrition was about 17% per year (Lipps, 2007). The primary interview mode was CATI (Computer Assisted Telephone Interviewing). The analytical sample for this paper was defined as respondents who participated at least twice in a network module of the panel while being 55 years old or older. Respondents were selected who were born between 1920 and 1960 (*N* = 5,585).

### Network Measures

The network module was held annually from 1999 to 2013 and every 3 years since then (15 waves of data). Respondents were asked with how many neighbors they were on good terms with and close to. Similarly, respondents were asked how many good and close friends they had. The numbers of neighbors and friends were used as outcomes. Values of 20 and higher were uncommon (fewer than 3%) and truncated to avoid outlier effects. If people said they had no friends or close neighbors, the variables were coded 0.

Next, respondents were asked how often they had contact with neighbors and friends, including telephone contact. The answers were recorded as the number of times per month where values above 30 were truncated. Contact with neighbors or friends was recoded to zero if the respondent reported zero neighbors or friends.

Respondents were next asked about support. The questions were as follows: (a) If necessary, in your opinion, to what extent can these (neighbors/friends) provide you with practical help (this means concrete help or useful advice), if 0 means “*not at all*” and 10 “*a great deal*”? (b) To what extent can these neighbors/friends be available in case of need and show understanding, by talking with you for example? Respondents were instructed to also answer the question if currently no support was needed. The advantage of potential support measures is that they are not confounded by actual support needs. Moreover, even knowing that one could receive support if a need emerges positively affects well-being (Van der Poel, 1993).

To compare neighbors and friends, four extra variables were created: (a) the proportion of neighbors out of the total number of neighbors and friends, (b) the proportion of neighbor contacts out of the total number of neighbor and friend contacts, (c) the difference between practical support from neighbors and friends, and (d) the difference between emotional support from neighbors and friends. Some people reported neither friends nor neighbors (4%) or no contact with either (7.6%). These were recoded to a proportion of 0.5, indicating no preference for either friends or neighbors.

### Independent Variables

Three birth cohorts were distinguished and these can be observed in partly overlapping age ranges: cohort 1920–1934 from age 65 to 90, cohort 1935–1944 from age 55 to 84, and cohort 1945–1959 from age 55 to 74.

Marital status was distinguished into four categories: married, divorced, widowed, and never married. The group of divorcees includes people who were informally separated from a marital union. For the age groups analyzed here, unmarried cohabitation was uncommon and not considered as a separate variable.

A distinction was made between respondents who were employed and all others (retired, unemployed, or never worked). Employment interacted with gender. The main effects apply to men, the interactions inform us whether the effects are weaker or stronger for women than for men.

Education and income were used to measure SES. Educational categories were recoded to the average years of schooling per category following the specifications of the SHP. Logged equivalized household income after taxes adjusted for price changes was used.

To control for residential differences, a distinction was made between rural settings (rural commuter communes, mixed agricultural communes, and peripheral agricultural communes), urban settings (centers), and places in between (all others). In addition, the number of years the respondents lived in their current dwelling was controlled for (Volker & Flap, 2007).

### Design

**Figure 1.**
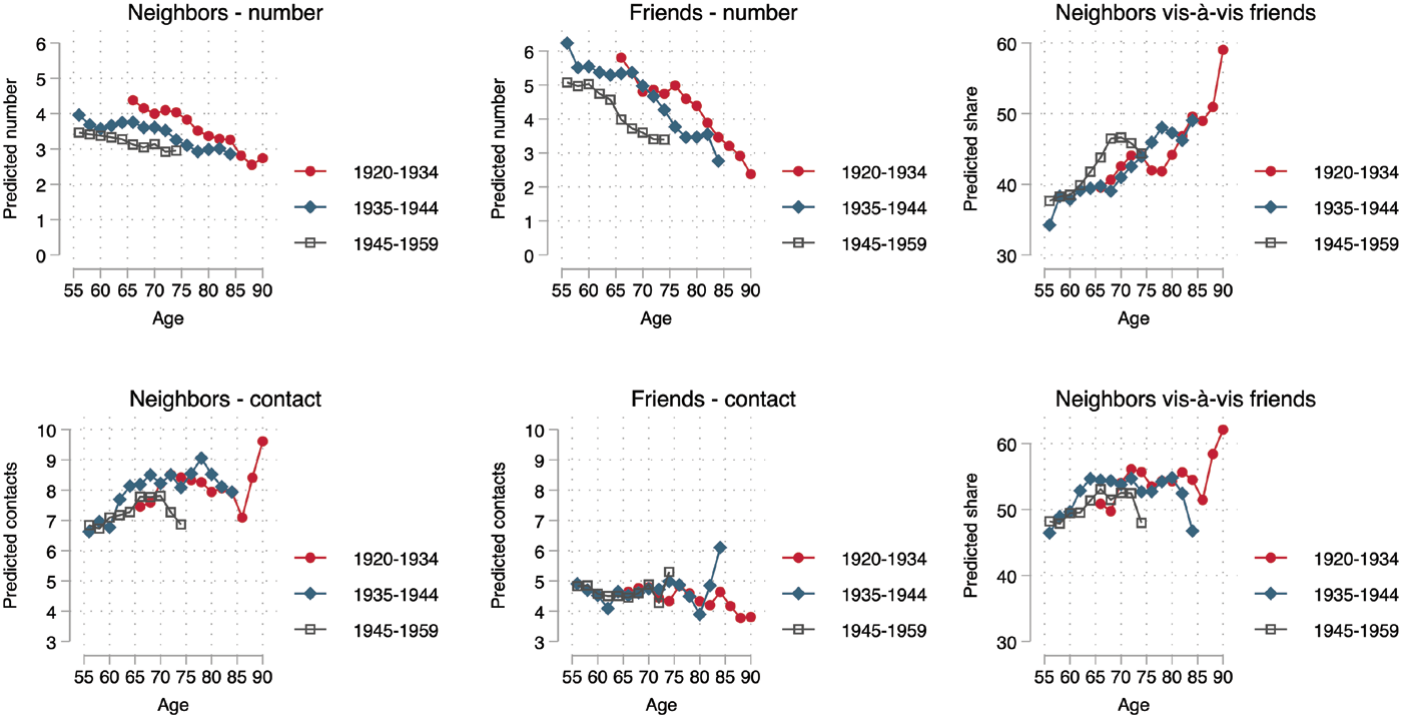
Changes in network size and contact by cohort. Based on fixed-effects models of Swiss Household Panel 1999–2019. Two-year age groups used.

**Figure 2.**
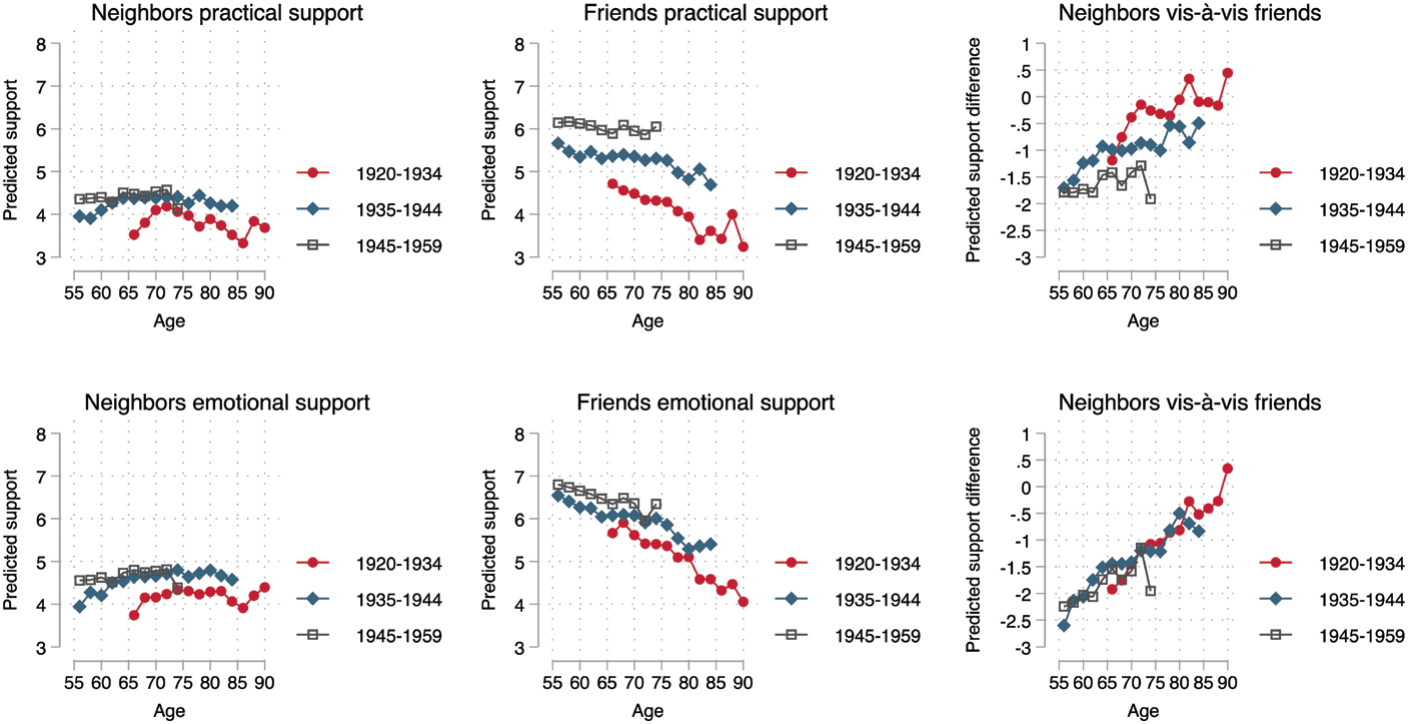
Changes in expected network support by cohort. Based on fixed-effects models of Swiss Household Panel 1999–2019. Two-year age groups used.

First, fixed-effects models that include age and cohort were estimated to test *H1* and *H2*. These models provide estimates of changes within persons as they age (Figures 1 and 2). Next, hybrid regression models were used to test *H3–H5*. These models allow us to simultaneously estimate the effects of changes in the independent variables within persons and the effects of differences between persons in the independent variables (Schunck, 2013; Uccheddu et al., 2019). For variables that change during the life course, such as age, marital status, and employment, the within-effects are of prime interest. For variables that are relatively or completely static at these ages, the between-effects are of prime interest. Hybrid models, especially when combined with interactions between age and the other independent variables, are similar to growth-curve models which have also been used in this literature (e.g., Shaw et al., 2007). In Supplementary Table 1, random-effects, fixed-effects, and growth curve models are compared for the interactions by age and marital status. The interaction effects are the same across these models.

### Missing Values

The proportion and difference variables were missing when one of the two elements was missing (Table 1). For the independent variables, only income had a substantial number of missing values (6.5%). For this reason, it was decided to impute income only. Income was imputed by first taking the income in a prior wave or the wave before the prior wave, and if not available, the average income of the respondent across valid waves. The regression models in Tables 2 and 3 were estimated on one sample for which all dependent and independent variables were defined. The number of persons in the models was *N* = 5,385 which is a reduction of 200 persons compared to the original sample.

**Table 1.** Summary Statistics of Variables in the Person-period File: Means for Continuous and Proportions for Dichotomous Variables

Variable	*N*	Mean	*SD*	Min	Max	Skewness
Women	33,047	0.567		0	1	−0.272
Age	33,047	66.8	8.1	55	90	0.53
Date of birth: Year	33,047	1,941.4	8.9	1,920	1,959	−0.261
Married	33,037	0.665		0	1	−0.7
Divorced	33,037	0.131		0	1	2.182
Widowed	33,037	0.136		0	1	2.126
Never married	33,037	0.068		0	1	3.442
Education	33,047	12.99	2.89	9	19	0.706
Household income (log)	32,773	10.67	0.68	0	12.28	−5.287
Retired/non-employed	33,047	0.604		0	1	−0.425
Years in current house	32,728	23.8	15.6	0	60	0.434
Rural residence	32,996	0.159		0	1	1.87
Medium residence	32,996	0.576		0	1	−0.309
Urban residence	32,996	0.265		0	1	1.065
Neighbors: number	32,733	3.497	3.596	0	20	2.016
Friends: number	32,678	4.745	3.969	0	20	1.85
Share of neighbors	32,436	41.5	27.5	0	100	0.225
Neighbors: contact frequency	32,447	7.678	9.123	0	30	1.198
Friends: contact frequency	32,526	4.635	5.988	0	30	2.469
Share of neighbor contact	32,042	51.9	34.3	0	100	−0.293
Neighbors: practical support	32,351	4.226	3.471	0	10	0.005
Friends: practical support	32,287	5.356	3.368	0	10	−0.464
Neighbors–friends support	31,765	−1.116	4.127	−10	10	0.023
Neighbors: emotional support	32,330	4.493	3.585	0	10	−0.098
Friends: emotional support	32,431	6.086	3.383	0	10	−0.778
Neighbors–friends support	31,856	−1.58	4.300	−10	10	0.028

*Note*: Source: Swiss Household Panel data 1999–2019.

**Table 2.** Hybrid Models of Network Size and Contact Frequency: Effects of Differences Between Persons (Between-Effects) and Changes Within Persons (Within-Effects)

Variable	(1)	(2)	(3)	(4)	(5)	(6)
	Neighbors number	Friends number	Share of neighbors	Neighbors contact	Friends contact	Share of neighbor contacts
Born “35–44” vs “20–34” (between)	−0.266*	−0.116	−0.178	0.193	0.478*	−1.619
	(.022)	(.395)	(.842)	(.546)	(.023)	(.160)
Born “45–59” vs “20–34” (between)	−0.651*	−1.019*	2.019**	−0.198	0.657*	−3.736*
	(.000)	(.000)	(.066)	(.612)	(.011)	(.008)
Women (between)	−0.306*	−0.229**	−1.885*	0.505	0.609*	−2.429*
	(.008)	(.090)	(.034)	(.111)	(.003)	(.034)
Age (within)	−0.047*	−0.099*	0.445*	0.034*	−0.019*	0.133*
	(.000)	(.000)	(.000)	(.006)	(.019)	(.005)
Age squared (within)	−0.001*	−0.002*	0.005*	−0.003*	−0.000	−0.009*
	(.000)	(.000)	(.033)	(.002)	(.665)	(.007)
Married → divorced (within)	0.226	−0.020	−1.123	−0.523	0.788*	−4.807*
	(.297)	(.927)	(.505)	(.340)	(.028)	(.023)
Married → widowed (within)	0.001	0.035	−0.665	0.215	1.454*	−2.375**
	(.993)	(.804)	(.536)	(.540)	(.000)	(.079)
Married → never married (within)^a^	−1.075**	−0.551	−2.625	−2.027	1.203	−13.274*
	(.073)	(.368)	(.573)	(.182)	(.226)	(.024)
Divorced vs married (between)	−0.378*	−0.130	−2.924*	0.130	1.863*	−7.009*
	(.000)	(.283)	(.000)	(.647)	(.000)	(.000)
Widowed vs married (between)	0.133	−0.122	1.171	1.096*	1.498*	−0.803
	(.253)	(.373)	(.190)	(.001)	(.000)	(.488)
Never married vs married (between)	−0.403*	−0.097	−2.593*	0.021	1.892*	−6.703*
	(.002)	(.533)	(.010)	(.954)	(.000)	(.000)
Education (between)	0.047	0.285*	−1.426*	0.050	−0.049	0.183
	(.182)	(.000)	(.000)	(.609)	(.450)	(.606)
Household income (between)	0.008	0.318*	−1.740*	−0.247*	−0.108	−0.558
	(.855)	(.000)	(.000)	(.034)	(.159)	(.187)
Employed → retired (within)	0.040	−0.287*	1.399*	0.399**	−0.146	1.907*
	(.647)	(.001)	(.039)	(.070)	(.311)	(.025)
× female	0.103	0.400*	−0.698	0.020	0.489*	−1.040
	(.353)	(.000)	(.418)	(.944)	(.008)	(.337)
Retired vs employed (between)	0.160	0.105	1.314	1.492*	−0.104	3.955*
	(.252)	(.519)	(.221)	(.000)	(.680)	(.004)
× female	−0.199	−0.202	−0.108	−1.434*	0.199	−2.607
	(.222)	(.289)	(.931)	(.001)	(.499)	(.107)
Constant	4.315*	5.240*	44.944*	7.656*	2.644*	60.423*
	(.000)	(.000)	(.000)	(.000)	(.000)	(.000)
*N* person-waves	30,459	30,459	30,459	30,459	30,459	30,459
*N* persons	5,385	5,385	5,385	5,385	5,385	5,385
Chi-squared	368.6	1,029.5	681.9	170.4	367.3	401.1
Rho (empty model)	0.300	0.391	0.301	0.350	0.367	0.317

*Notes*: Source: Swiss Household Panel data 1999–2019.

*p* Values in parentheses. Controlled for urbanization and tenure in residence. Between-effects of age and within-effects of income not printed.

^a^Effect refers to the transition from never married to married (after taking the negative).

*p < .05.

**p < .10.

**Table 3.** Hybrid Models of Expected Practical and Emotional Support: Effects of Differences Between Persons (Between-effects) and Changes Within Persons (Within-effects)

Variable	(1)	(2)	(3)	(4)	(5)	(6)
	Neighbors practical support	Friends practical support	Support neighbors–support friends	Neighbors emotional support	Friends emotional support	Support neighbors–support friends
Born “35–44” vs “20–34” (between)	0.316*	0.915*	−0.569*	0.363*	0.561*	−0.188
	(.012)	(.000)	(.000)	(.005)	(.000)	(.189)
Born “45–59” vs “20–34” (between)	0.424*	1.481*	−1.020*	0.515*	0.867*	−0.344**
	(.006)	(.000)	(.000)	(.001)	(.000)	(.051)
Female (between)	0.446*	0.821*	−0.367*	0.431*	1.107*	−0.670*
	(.000)	(.000)	(.006)	(.001)	(.000)	(.000)
Age (within)	−0.004	−0.027*	0.022*	0.010*	−0.045*	0.055*
	(.346)	(.000)	(.000)	(.036)	(.000)	(.000)
Age squared (within)	−0.001*	−0.001*	0.000	−0.001*	−0.001*	0.000
	(.000)	(.000)	(.838)	(.002)	(.000)	(.304)
Married → divorced (within)	0.242	0.668*	−0.426**	0.237	0.666*	−0.429
	(.229)	(.000)	(.095)	(.254)	(.000)	(.106)
Married → widowed (within)	0.609*	0.585*	0.023	0.402*	0.551*	−0.149
	(.000)	(.000)	(.887)	(.002)	(.000)	(.379)
Married → never married (within)^a^	−0.495	0.281	−0.777	−0.589	−0.331	−0.258
	(.374)	(.582)	(.272)	(.306)	(.528)	(.726)
Divorced vs married (between)	−0.149	0.316*	−0.471*	−0.164	0.383*	−0.551*
	(.188)	(.003)	(.000)	(.159)	(.000)	(.000)
Widowed vs married (between)	0.357*	0.228**	0.122	0.352*	0.188	0.153
	(.005)	(.061)	(.369)	(.008)	(.125)	(.286)
Never married vs married (between)	−0.137	0.488*	−0.627*	−0.222	0.422*	−0.643*
	(.342)	(.000)	(.000)	(.136)	(.002)	(.000)
Education (between)	0.101*	0.193*	−0.092*	0.051	0.202*	−0.149*
	(.009)	(.000)	(.028)	(.206)	(.000)	(.001)
Household income (between)	0.156*	0.279*	−0.129*	0.112*	0.296*	−0.190*
	(.001)	(.000)	(.010)	(.018)	(.000)	(.000)
Employed → retired (within)	−0.040	−0.149*	0.109	−0.049	−0.104	0.054
	(.622)	(.045)	(.289)	(.554)	(.175)	(.612)
× female	0.097	0.173**	−0.076	0.044	0.058	−0.014
	(.346)	(.066)	(.560)	(.677)	(.549)	(.919)
Retired vs employed (between)	0.276**	−0.114	0.392*	0.164	−0.235	0.404*
	(.068)	(.432)	(.016)	(.295)	(.109)	(.019)
× female	−0.206	−0.252	0.051	−0.217	−0.175	−0.042
	(.246)	(.137)	(.788)	(.236)	(.307)	(.834)
Constant	3.932*	3.919*	−0.004	4.272*	4.914*	−0.641*
	(.000)	(.000)	(.985)	(.000)	(.000)	(.002)
*N* person−waves	30,459	30,459	30,459	30,459	30,459	30,459
*N* persons	5,385	5,385	5,385	5,385	5,385	5,385
Chi-squared	277.0	968.9	546.3	246.6	858.1	563.5
Rho (empty model)	0.405	0.471	0.312	0.406	0.447	0.317

*Notes*: Source: Swiss Household Panel data 1999–2019.

*p* Values in parentheses. Controlled for urbanization and tenure in residence. Between-effects of age and within-effects of income not printed.

^a^Effect refers to the transition from never married to married (after taking the negative).

*p < .05.

**p < .10.

### Findings

As can be seen in Table 1, the proportion of neighbors out of the total number was 0.41 (*SD* = 0.28) and the proportion of neighbor contacts was 0.52 (*SD* = 0.36). In other words, the average older person had more friends than neighbors in the network but about the same amount of contact with the two network segments. Practical support was higher on average for friends than for neighbors and this difference was even larger for emotional support. The skewness measures indicate that the sizes of the subnetworks as well as the contact frequencies are positively skewed, with many people having a few neighbors or friends and a few having large numbers.

### Changes

Figure 1 presents predictions from the fixed-effects models for the size of the network and the amount of contact separately for each cohort. For all cohorts, Figure 1 shows a decline in network size with age. The decline was present for both neighbors and friends but considerably steeper for friends. As a consequence, the share of friends declined with age. At age 55, about 35% of this segment of the nonkin network consisted of neighbors, at age 80, the majority were neighbors (50%–60%).

When looking at contacts, the trends were partly different and partly similar. Contacts with neighbors increased with age. For friends, there was no change in the amount of contact with age but because the number of friends declined, this also suggests a trend toward more contact with a shrinking pool of friends. When comparing the two trends, the conclusion is the same as it was for network size: the share of neighbor contact increased with age. At the highest ages in the data, people had more contact with neighbors than with friends.

Figure 2 shows the margins for expected practical and emotional support. For both types of support, there was no change in the degree of support from neighbors with age. For friends, there was a decline with age in how important they are perceived to be as a source of support. The decline in the importance of friends was surprisingly similar for practical and emotional support. Combining the two age trends shows that neighbors became relatively more important for support than friends as people aged. Positive values indicate an advantage for neighbors, negative values indicate an advantage for friends. The graph shows that the age pattern was from “friend dominance” at earlier ages toward parity, as the values were about zero at the highest observed ages.

In sum, the age trends confirm *H1a* and show that the shift from friends toward neighbors is a general tendency, which applies to all network indicators, including emotional support, thereby refuting *H1b*.

### Differentials

The two figures also show how the cohorts differ. There was no consistent pattern in the cohort effects. Keeping age constant, recent cohorts had relatively more neighbors but less contact with them. Moreover, recent cohorts received less practical support and the same amount of emotional support from neighbors vis-à-vis friends. The graphs make clear that the only convincing pattern emerging is the lower level of practical support from neighbors compared to friends in recent cohorts (Figure 2). This finding is in line with a historical shift from neighbors toward friends, but as it applies to only one of the four indicators, the evidence for *H2* is weak.

There were significant effects of changes and differentials in marital status. For most network measures, it was found that divorce was associated with an increased share of friends vis-à-vis neighbors. This conclusion applies to the between-effects and the more stringent within-effects. The effects largely emerged because friends were or became more prevalent for divorced respondents without neighbors being or becoming less prevalent (for contact and support). There were also significant widowhood effects. The between and within-effects indicate that widowhood was associated with more contact and support from neighbors and friends. For support, the effects were similar for neighbors and friends but for contact, the effects were stronger for friends. After widowing, older adults had relatively more contact with friends than with neighbors. In sum, neighbors tend to be dominant for married people while friends tend to be dominant for unmarried people. This confirms *H3a*.

After retirement, men had a smaller number of friends and more contact with neighbors. As a result, the share of neighbors and friends in terms of size and contact shifted toward neighbors after retirement. For support, the within-effects were small and insignificant. This provides partial support for *H4a*. For the size of the network and contact frequency, we also observe gender interactions. The decline in the number of friends after retirement for men was absent for women, as the interaction showed. The main effect for men on the number of friends was −0.287 and the interaction was *b* = 0.400, implying an effect for women on the number of friends of *b* = −0.287 + 0.400 = +0.113. Similarly, the share of contact with neighbors increased for men (*b* = 1.907) but less so for women (*b* = 1.907 − 1.040 = 0.877. The between effects were generally stronger and more often significant. The findings thereby provide support for *H4b*. Retirement for men is associated with a shift from friends to neighbors.

The share of neighbors vis-à-vis friends also depended on SES. Here, we focus mostly on the between effects. Older adults with more income and education had relatively more friends than neighbors and received more practical and emotional support from friends than from neighbors, as predicted by *H5a*. Interestingly, there was little socioeconomic differentiation in neighborhood ties itself; it was primarily because friends were more common and supportive in higher SES groups that the relative outcomes were affected.

### Differential Change?

To assess if the differentials just discussed changed with age, additional fixed-effect models were estimated. In the first model, age interactions were added for marital status without other control variables. Because the interactions of marital status and the squared term of age were insignificant, only the linear term interacted with age. The interaction of age and divorce was *b* = 0.458 (*p* < .01) for the relative size of the neighbor network, *b* = 0.543 (*p* < .01) for the relative amount of contact with neighbors, *b* = 0.060 (*p* < .01) for the difference in expected practical support, and *b* = 0.055 (*p* < .01) for the difference in expected emotional support. For widowhood (vs the married state), we found significant interactions of *b* = 0.319 (*p* < .01) for the share of contact with neighbors, *b* = 0.029 (*p* = .04) for the difference in practical support, and *b* = 0.027 (*p* = .06) for the difference in emotional support.

**Figure 3.**
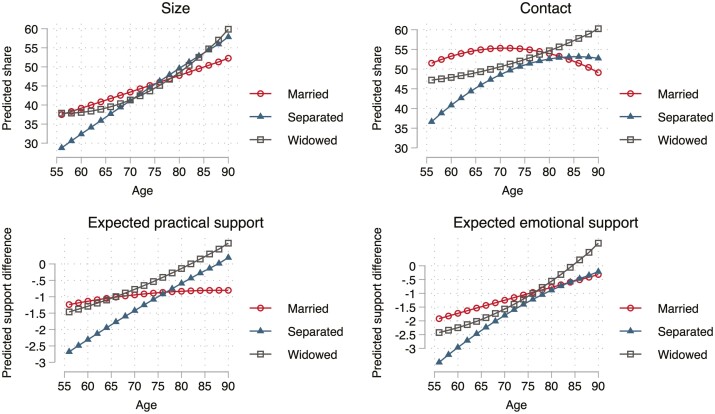
Interactions of age and marital status for difference between neighbors and friends. Based on fixed-effects models of Swiss Household Panel 1999–2019. Models estimated separately for marital status categories.

Figure 3 shows margins by age and marital status to illustrate the interactions. The figures show that for a divorced person at the beginning of the age interval, friends were clearly more common and supportive than neighbors. However, the gap between married and divorced persons declined with age, as hypothesized in (*H3b*). At very old ages, neighbors actually became slightly more prevalent than friends for divorced persons. A similar pattern emerges for widowhood. At the early ages, widowed persons were relatively more oriented toward friends than were married persons but the increase in the prevalence of neighbors with age was stronger for widowed persons, closing the gap with married persons. Another way of interpreting the interactions is by pointing out that the previously observed shift from friends toward neighbors is stronger for widowed and divorced people.

In a similar way, we tested whether there were interactions between age and education in a fixed-effects model with only age, cohort, and education. In contrast to hypothesis *H5b*, we found no significant interactions.

## Conclusion and Discussion

Using long-term panel data from one national context, and information on the size of networks, the frequency of contact, and the perceived degree of support, this study showed that as people become older, neighbors become more important vis-à-vis friends. Even though the sizes of both network segments decline with age, in line with previously found personal network shrinkage in later life, the number of friends declines faster, making neighbors more important than friends at the oldest ages. The size of the nonkin network declines whereas the amount of contact does not, suggesting growing intensity of ties with age. More importantly, however, contact with neighbors increases with age, whereas contact with friends does not. As a result, neighbors are becoming more important as a source of contact than friends when people grow older. The same conclusion is reached for support, with neighbors becoming relatively more important with age. However, it is only at very high ages that neighbors are more important than friends in terms of network size and contact frequency, and at these ages, expected support levels are similar for the two network segments.

In contrast to expectations, suggested by socioemotional selectivity theory, the growing importance of neighbors over friends applies not only to practical support and contact but also to emotional support. This somewhat unexpected finding can be interpreted in terms of spillover effects. Contact and practical support exchange provide opportunities for sharing personal concerns and intimacy, and this may foster the exchange of emotional support. The shift away from friends toward neighbors does therefore not necessarily imply a decline in the strength of ties and an increase in more instrumental ties.

Differentials in the comparison between neighbors and friends were also found but there was mixed evidence for differential change. For married people, people who retired, and people with a lower SES, neighbors are more important than friends. Effects of marital status and retirement persisted in a within-person change design. There was a decline in marital status differences with age, making neighbors eventually also more important for unmarried people. It was further expected that mortality differences by SES would harm the friendship network of people with a lower SES more with age, but the SES gap in the importance of neighbors vis-à-vis friends was stable.

Cohort differences could provide clues about historical changes in social life. Most trend studies have focused on overall network indicators or on specific segments of the network, addressing the question of a decline in nonkin ties more generally (Suanet et al., 2013). The current study aimed to test the claim of a shift toward more individualized and voluntary relationships. The comparison of neighbors and friends is informative, recognizing that neighbors and neighborhoods are also a matter of choice, at least indirectly. The evidence does not support any trend theory. Recent cohorts rely less on practical support from neighbors versus friends but cohort effects for the other outcomes are small or contrary (for size).

A number of limitations need to be discussed. First, networks were not measured using ego-centered networks that have been used in the network literature. The more global network measures used here are collected efficiently and repeatedly over a long period of time in a national panel survey. The detailed network measures used elsewhere often take much interview time and are therefore rarely collected in long-running national panel surveys.

Second, the findings apply to one national context. Switzerland has an aging population like other Western European countries with a relatively high life expectancy (Eurostat, 2020). The vast majority of people 65+ live alone or in a couple, about 90% (Eurostat, 2020). The perceived quality of the local area where older people live is about average and relatively few older people in Switzerland experience material deprivation (Eurostat, 2020).

Third, the study did not consider ties to family, colleagues, and association members. Future studies can extend the current design to incorporate these ties but the comparison of neighbors vis-à-vis friends will be unaffected. Relatedly, it needs to be recognized that the two network segments may overlap. People can end up living close to someone who was already a friend and a neighbor can eventually become a friend. It is not known how people categorize these forms of overlap. The SHP first asked respondents about neighbors with whom they were close and then asked about friends. As a result, it is plausible that a close neighbor whom the respondent could also regard as a friend was not included in the friendship measures.

The general conclusion of this study is that there is a rising importance of neighbors vis-à-vis friends with age. For network size and support, this shift is driven by a decline in the importance of friends without a corresponding decline in neighbors. For contact, there was an age-related increase for neighbors as well. In general, our finding suggests that proximity principles help older people to stabilize their network, in line with notions about aging in place (Wiles et al., 2012) and the persisting importance of geography for social life more generally (Fischer, 2011; Wellman et al., 2001). The importance of neighbors in old age can be relevant for designing care and housing policies. The desire of older people to live independently in the neighborhood in which they lived for years matches the findings in this paper. A recent study of older people’s housing preferences emphasizes the importance of their own home in old age and concludes that to realize such preferences, programs are needed that include local aging service resources (Gibson et al., 2023). The current study emphasizes the necessity of combining local care services with neighborhood integration to enhance healthy aging.

## Supplementary Material

gbae108_suppl_Supplementary_Table
