# Supplementary material for: Comparing Neighbors and Friends in Age-Related Network Changes
Source: J Gerontol B Psychol Sci Soc Sci. 2024 Jun 29;79(9):gbae108. doi: 10.1093/geronb/gbae108 (PMC11304952; doi:10.1093/geronb/gbae108)
Supplement: gbae108_suppl_Supplementary_Table [file gbae108_suppl_supplementary_table.docx]

***The Journals of Gerontology, Series B: Psychological Sciences and Social Sciences* Supplementary Material: Kalmijn. Comparing Neighbors and Friends in Age-Related Network Changes.**

Supplementary Table 1. Random-effects (re), fixed-effects (fe), and growth curve (gc) models with age interactions

| Variable | Share of neighbors in network | | | Share of neighbor contacts | | |
| --- | --- | --- | --- | --- | --- | --- |
|  | re | fe | gc | re | fe | gc |
| Age | .395^*^ | .426^*^ | .391^*^ | .138^*^ | .118^*^ | .150^*^ |
|  | (.000) | (.000) | (.000) | (.001) | (.014) | (.001) |
| Age # Age | .003 | .004 | .004 | -.012^*^ | -.012^*^ | -.013^*^ |
|  | (.199) | (.133) | (.131) | (.000) | (.000) | (.000) |
| Divorced | -3.429^*^ | -.540 | -3.570^*^ | -7.318^*^ | -4.449^*^ | -7.384^*^ |
|  | (.000) | (.752) | (.000) | (.000) | (.039) | (.000) |
| Widowed | .373 | -1.201 | .371 | -2.161^*^ | -4.106^*^ | -2.088^~^ |
|  | (.638) | (.333) | (.670) | (.032) | (.009) | (.056) |
| Female | -.619 |  | -.527 | -3.862^*^ |  | -3.792^*^ |
|  | (.246) |  | (.335) | (.000) |  | (.000) |
| Born ‘35-44 vs ‘20-34’ | 1.306^~^ |  | 1.244 | -.468 |  | -.402 |
|  | (.095) |  | (.105) | (.639) |  | (.684) |
| Born ‘45-59 vs ‘20-34 | 3.900^*^ |  | 4.069^*^ | -1.871^~^ |  | -1.713^~^ |
|  | (.000) |  | (.000) | (.069) |  | (.097) |
| Divorced # Age | .345^*^ | .458^*^ | .333^*^ | .372^*^ | .543^*^ | .342^*^ |
|  | (.000) | (.000) | (.000) | (.000) | (.000) | (.001) |
| Widowed # Age | .101 | .139 | .104 | .152^~^ | .319^*^ | .147 |
|  | (.159) | (.131) | (.199) | (.094) | (.006) | (.151) |
| Constant | 40.679^*^ | 41.720^*^ | 40.572^*^ | 57.270^*^ | 54.370^*^ | 57.228^*^ |
|  | (.000) | (.000) | (.000) | (.000) | (.000) | (.000) |
|  | Practical support neighbors - friends | | | Emotional support neighbors - friends | | |
|  | re | fe | gc | re | fe | gc |
| Age | .020^*^ | .020^*^ | .021^*^ | .047^*^ | .050^*^ | .048^*^ |
|  | (.000) | (.001) | (.000) | (.000) | (.000) | (.000) |
| Age # Age | -.000 | -.000 | -.000 | .000 | .000 | .000 |
|  | (.658) | (.636) | (.561) | (.825) | (.689) | (.936) |
| Divorced | -.546^*^ | -.422 | -.553^*^ | -.646^*^ | -.386 | -.648^*^ |
|  | (.000) | (.103) | (.000) | (.000) | (.151) | (.000) |
| Widowed | -.022 | -.141 | .002 | -.037 | -.293 | -.028 |
|  | (.857) | (.453) | (.988) | (.771) | (.133) | (.843) |
| Female | -.203^*^ |  | -.212^*^ | -.514^*^ |  | -.511^*^ |
|  | (.011) |  | (.008) | (.000) |  | (.000) |
| Born ‘35-44 vs ‘20-34’ | -.538^*^ |  | -.512^*^ | -.028 |  | -.007 |
|  | (.000) |  | (.000) | (.825) |  | (.952) |
| Born ‘45-59 vs ‘20-34 | -.985^*^ |  | -.960^*^ | -.117 |  | -.105 |
|  | (.000) |  | (.000) | (.364) |  | (.415) |
| Divorced # Age | .053^*^ | .060^*^ | .051^*^ | .041^*^ | .055^*^ | .039^*^ |
|  | (.000) | (.000) | (.000) | (.000) | (.000) | (.002) |
| Widowed # Age | .028^*^ | .029^*^ | .027^*^ | .021^~^ | .027^~^ | .022^~^ |
|  | (.010) | (.038) | (.028) | (.059) | (.061) | (.088) |
| Constant | -.291^*^ | -.977^*^ | -.306^*^ | -1.082^*^ | -1.423^*^ | -1.095^*^ |
|  | (.008) | (.000) | (.005) | (.000) | (.000) | (.000) |

Source: Swiss Household Panel data 1999-2019. P-values in parentheses. Age centered around the mean. Never-married category excluded.

^~^ *p* < 0.10, ^*^ *p* < 0.05.
